# Sensory Cortical Plasticity Participates in the Epigenetic Regulation of Robust Memory Formation

**DOI:** 10.1155/2016/7254297

**Published:** 2016-01-03

**Authors:** Mimi L. Phan, Kasia M. Bieszczad

**Affiliations:** Psychology Department, Behavioral & Systems Neuroscience, Rutgers, The State University of New Jersey-New Brunswick, Piscataway, NJ 08854, USA

## Abstract

Neuroplasticity remodels sensory cortex across the lifespan. A function of adult sensory cortical plasticity may be capturing available information during perception for memory formation. The degree of experience-dependent remodeling in sensory cortex appears to determine memory strength and specificity for important sensory signals. A key open question is how plasticity is engaged to induce different degrees of sensory cortical remodeling. Neural plasticity for long-term memory requires the expression of genes underlying stable changes in neuronal function, structure, connectivity, and, ultimately, behavior. Lasting changes in transcriptional activity may depend on epigenetic mechanisms; some of the best studied in behavioral neuroscience are DNA methylation and histone acetylation and deacetylation, which, respectively, promote and repress gene expression. One purpose of this review is to propose epigenetic regulation of sensory cortical remodeling as a mechanism enabling the transformation of significant information from experiences into content-rich memories of those experiences. Recent evidence suggests how epigenetic mechanisms regulate highly specific reorganization of sensory cortical representations that establish a widespread network for memory. Thus, epigenetic mechanisms could initiate events to establish exceptionally persistent and robust memories at a systems-wide level by engaging sensory cortical plasticity for gating *what* and *how much* information becomes encoded.

## 1. Introduction

Nearly four decades of research have established that life-long learning alters cortical representations of the experienced sensory world—sensory cortical representations are plastic. However, sensory cortical plasticity not only underlies sensory processing* per se*, but also is relevant for the learning of new information by facilitating neural processes for encoding, storing, and remembering informative links between sensory events and their outcomes. That sensory cortical representations change with experience could answer the fundamental question:* How does actual information become part of the contents of memory?*

The sensory cortices lie at a unique junction between perceptual and cognitive functions because plasticity even in early sensory areas can induce selective behavioral changes in signal detection, discrimination, categorization, learning, and memory, or in some combination [1–3]. These functions act on information about sensory signals and their behaviorally relevant physical features, thus providing a window for sensory cortex to enable content in memory with the same perceptual vividness of an initial experience. A fundamental issue is to identify the mechanisms of experience-dependent sensory cortical plasticity that support learning and memory in adult brains—especially those mechanisms that lead to behaviorally adaptive outcomes throughout life.

It is important to note that learning experiences do not always produce sensory cortical plasticity, nor do they always lead to veridical memory or behaviorally adaptive outcomes for cognition or for perception. However, the induction of sensory cortical plasticity appears to occur when a learning experience does lead to the formation of a strong specific memory. This plasticity likely depends on the synergistic engagement of many neuromodulatory and molecular events to induce the changes in neural circuits that ultimately underlie memory formation at various timescales [4–7].

Some memories are transient with immediate or short-term utility, while others can last a lifetime. What are the neural mechanisms that can set the timescale of memory? An initial answer to this question has been identifying gene expression as a necessary catapult between short-term and long-term memory processes [8]. Recent findings have shown that epigenetic mechanisms that alter gene expression may impact adult sensory cortical plasticity, memory, and sensory discrimination ability [9–11]. Thus epigenetic modulation could create a permissive state for learning to transform experiences into long-term memories by facilitating encoding in sensory cortical processes. The goal of this review is to highlight the potential for epigenetic mechanisms that control gene expression to set the threshold of induction for robust and persistent memories by enabling information encoding in sensory cortices.

## 2. Epigenetic Mechanisms Controlling Neuroplasticity in the Adult Brain

Epigenetics can be defined as the posttranslational physical marking of proteins or of DNA itself in ways that modify the conformation of chromatin within the cell nucleus. Proteins called histones aid in packaging DNA from a loose strand into a densely packed arrangement of DNA wound around pairs of histones that together form an octamer called a nucleosome, which is the building block of the eventual higher-order structure of chromatin. Specialized enzymes can selectively target modifications to DNA, or to lysine residues on the tail-like structures of histone subunits, and even rearrange nucleosomes within selective genomic regions to permit stable changes in transcription that establish long-lasting effects for neuroplasticity and—ultimately—behavior. Of the behavioral epigenetic mechanisms known to act dynamically in adult neurons (DNA methylation, histone acetylation or methylation, histone variance, chromatin remodeling, microRNAs, and nucleosome remodeling [16–18]),* histone acetylation* is highlighted here to underscore its apparent importance for dynamic adaptations to sensory-cognitive functions and underlying experience-dependent sensory cortical plasticity.

Histone deacetylases (HDACs) and their counterpart enzymes, the histone acetyltransferases (HATs), effectively remove or add acetyl groups to lysine residues on histone tails in a way that represses or enables gene expression, respectively. An HDAC called HDAC3 (in class I family of HDACs 1, 2, 3, and 8) has been uniquely described as a “molecular brake pad” on memory formation [19–21]. Briefly stated, the molecular brake pad hypothesis predicts that HDAC3 can occupy the promoters of genes that are critical for the consolidation of memory to “put the brakes” on memory formation [19]. The enzymatic action of HDAC3 in the region of these promoters suppresses the ability of transcriptional machinery to become recruited for expression of local genes. Hence, HDAC3 in particular has been called a* molecular brake* on long-term memory formation.

**Figure 1:**
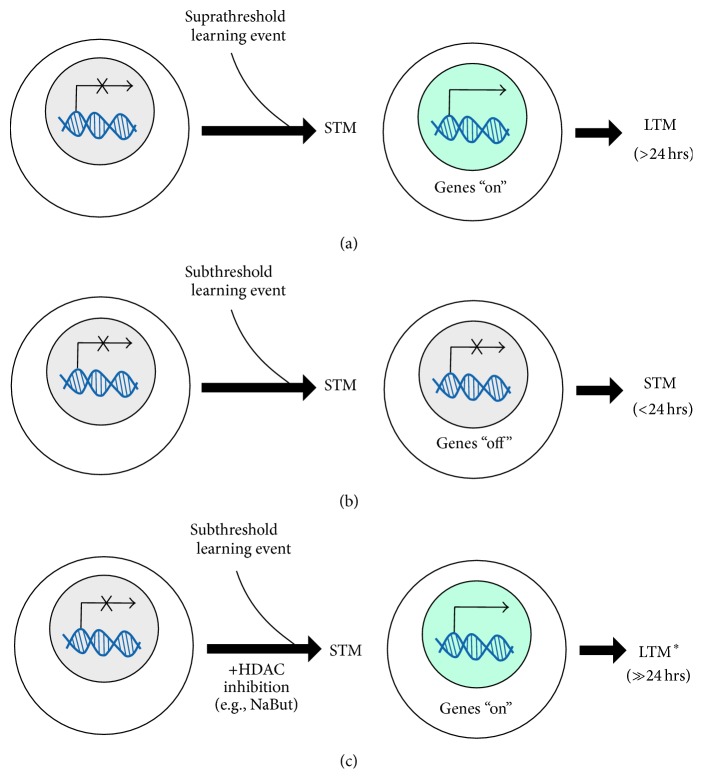
Long-term memory formation at the neuronal level.* A mechanistic simplification of how behavioral epigenetics exert molecular control of gene expression for memory formation.* (a) At a neuronal level, memory consolidation requires gene expression (genes “on”), which “suprathreshold” learning events can naturally activate to result in long-term memory (LTM: long-term memory, e.g., >24 hrs). Note that this depiction is an oversimplification showing only the requirement for gene expression, however which genes are activated, the magnitude of their expression, and also the temporal dynamics of transient or sustained expression are also factors.* Circles represent a single neuron with gene expression events occurring inside the cell nucleus (shaded).* (b) In contrast, “subthreshold” learning events fail to induce gene expression for memory consolidation and therefore produce short-term memory (STM, e.g., <24 hrs) but not long-term memory (LTM, e.g., >24 hrs). (c) A special case exists if the threshold for induction of a long-term memory is lowered by an epigenetic manipulation like HDAC inhibition (e.g., the administration of sodium butyrate (NaBut), a nonselective general class I HDAC inhibitor). In this scenario, the “subthreshold” experience can be made to produce long-term memory. Moreover, the memory that forms is robust and persistent at longer timescales beyond the point at which natural memory would fail (see [12, 13]).* Asterisk indicates enhanced LTM.*

Much has been revealed using a linear view of memory as the conceptual platform for understanding behavioral epigenetic influences of molecular control of gene expression. For example, so-called “subthreshold” learning events produce short-term memory (STM, e.g., <24 hrs) and not long-term memory (LTM, e.g., >24 hrs). Failure of LTM is most often explained by a failure to induce memory consolidation, which requires gene expression [8, 22–24] (Figures 1(a) and 1(b)). This linear view outlines a direct path for memory formation in a general progression where experiences transform from short- (STM, very weak; timescales of minutes), to immediate- (ITM, weak; timescales of several hours), and, with consolidation, to long-term (LTM, strong; timescales greater than 24 hours) and even life-long memory [25–34]. Studies investigating the role of histone acetylation for learning and memory in this framework have proposed that removing an HDAC molecular brake to increase acetylation at target sites on histones can effectively convert short-term into long-term memory by altering the threshold for mechanisms of memory consolidation [12, 35].

Stefanko et al. [12] introduced the initial hypothesis that epigenetic mechanisms regulate memory processes by lowering the threshold for the induction of a long-term memory. Thus, “subthreshold” experiences could be made to produce LTM with HDAC inhibition that enables gene expression. To test this hypothesis, they use standard novel object recognition (NOR) tasks in mice with administration of sodium butyrate (NaBut; a nonselective general class I HDAC inhibitor). NOR tasks consist of a training phase in which mice are allowed to explore an arena with two identical objects and a testing phase in which one of the familiar objects is replaced with a novel object. STM is often measured at 90 minutes after training and at 24 hours for LTM. Memory would be indicated if the animals recognized the novel object (by increased exploration time with the novel object), relative to the remembered object. In baseline assessments, the authors established that training with a 10-minute exposure to the objects was sufficient for LTM. Notably, 3 minutes of training exposure was not sufficient for the formation of LTM. However, mice administered with a single systemic injection of the nonselective HDAC inhibitor (NaBut) after 3 minutes of training could successfully discriminate the novel from the familiar object 24 hours later. Thus, HDAC inhibition transformed a learning event normally only inducted to STM (i.e., subthreshold from consolidation) to be encoded also in LTM. Therefore, HDAC inhibition (HDACi) enabled consolidation for LTM. This effect is interpreted as an HDACi-mediated increase in the strength of memory with respect to durability over time.

Following up on these experiments, McQuown et al. [13] determined that the HDAC effect on memory observed behaviorally could be obtained with a more selective pharmacological inhibitor (RGFP136) with enhanced selectivity for class I HDAC called HDAC3. Importantly, this study addressed whether HDAC effects on memory were task- and brain-region-specific. In addition to testing for object* recognition* memory (ORM) as Stefanko et al. did with their NOR task, mice in McQuown et al.'s study [13] were also tested for known hippocampally dependent object* location* memory (OLM) in which animals can recognize that one of the exposed objects at training has been moved to a new location during the retention test. Memory is indicated if mice increase exploration time with the moved object, relative to the undisturbed object [20]. An understanding of brain-region specificity of HDAC3 effects was made possible by focal deletion of HDAC3 in the dorsal hippocampus with bilateral intrahippocampal infusions of AAV-Cre recombinase in HDAC3-flox C57BL/6 mice weeks before subthreshold training (i.e., 3-minute duration of object exposure). Deleting HDAC3 in hippocampus resulted in the induction of long-term OLM but without effect to enable LTM for ORM. The animals all showed memory at the short-term time point for both location and recognition of the exposed objects as expected; however the treated animals were also able to achieve LTM but only for object* location*. The same result occurred with intrahippocampal infusions of the pharmacological HDAC3 inhibitor, RGFP136, in wild-type mice: the formation of long-term OLM but still no long-term ORM. Thus, a block of HDAC3 function in hippocampal neurons that are thought to be necessary for location memory was sufficient to induce LTM, and only for the location feature of the trained object. This provides evidence for HDAC3 function that is task- and brain-region specific. Therefore, the effect of HDACi to increase the strength of memory can be selective for the relevant information of the task, for example, for object identity versus object location. Moreover, the results suggest that populations of neurons with selectivity for each type of information are also preferentially engaged with HDAC action.

Brain region specificity was confirmed by the observation of accompanying relative increased acetylation of histone H4K8 and the concurrent upregulation of immediate early gene expression (e.g.,* c-fos* and* nr4a2*). These effects were evident only in the hippocampus and indicate a region-specific engagement of processes for plasticity related to learning about hippocampus-dependent spatial information. Similar findings in other tasks and with other class I HDAC inhibitors support the idea that removing an epigenetic “brake” on gene expression allows a permissive state for memory to consolidate from short- to long-term memory. Evidence from studies using other types of learned information, including spatial contexts associated with drugs of abuse, also supports the idea that HDAC inhibition engages plasticity in the selective brain regions or populations of neurons that are critical for task performance, such as the nucleus accumbens (NAc) for drug-related memory [35–39].

Taken together, these studies support at least two apparent roles of class I HDACs, and maybe in particular of HDAC3, in memory formation: (1) to modulate the strength of memory formation with respect to its durability over time, especially beyond 24 hours, and (2) to selectively act within neural brain regions (and neurons) that have behaviorally relevant information of the tasks to-be-remembered.

## 3. HDAC Inhibition Makes Memories That Outlast Those Naturally Formed

A curious anomaly exists in these seminal studies that established epigenetic mechanisms as critical regulators of memory formation. As noted, animals trained with the so-called “subthreshold” experiences will only form short-term memory that lasts less than 24 hours. If given an HDAC inhibitor systemically (e.g., a nonselective class I inhibitor like sodium butyrate or also RGFP936 or RGFP968 [39], or with more selective inhibitors with enhanced selectivity for HDAC3 like RGFP136 [13] and RGPF966 [9, 36] in particular brain regions) the animals will also form a LTM that does last 24 hours. However the effect lasts beyond this time point. The striking result across these studies is that LTM enabled by HDAC inhibition appears to outlast memories formed by natural* suprathreshold* training (Figure 1(c)). Additional experiments by Stefanko et al. [12] showed that LTM mediated by HDAC inhibition for subthreshold exposure (brief 3-minute training) can persist up to at least 7 days beyond the 24-hour limit of NOR retention observed after regular suprathreshold exposure (10-minute training in normal animals). Likewise, McQuown et al. [13] had similar results even with a single dose of the HDAC3-selective inhibitor (RGFP136): long-term OLM was enhanced and shown to last beyond the point at which natural long-term memory failed.

The mechanism for this relative increase in the robustness of memory achieved with HDAC inhibitors beyond the time points of natural memory formation has yet to be described. At the neuronal level, attempts have been made to identify the particular genes and the temporal dynamics of their expression that could produce a quantum change in the transcriptional landscape such that the coincident activation of families of genes produces opportunities for novel downstream gene products. Thus, one potential explanation for the robustness of long-term memory with HDACi is that prolonged gene expression dynamics, or the recruitment of families of genes for expression, produce a new genetic landscape that enables remarkable long-term potentiation and synaptic reweighting that stabilizes plasticity. Further downstream circuit-level influences of synaptic change could thereby push the system to its plausible physiological limits for robust changes in neuronal activity and, ultimately, behavior. Evidence for this possibility is beginning to emerge by following the unusual dynamics of immediate-early gene expression and genes that code for transcription factors, which can promote a further cascade of events for exceptional synaptic consequences [40–42].

**Figure 2:**
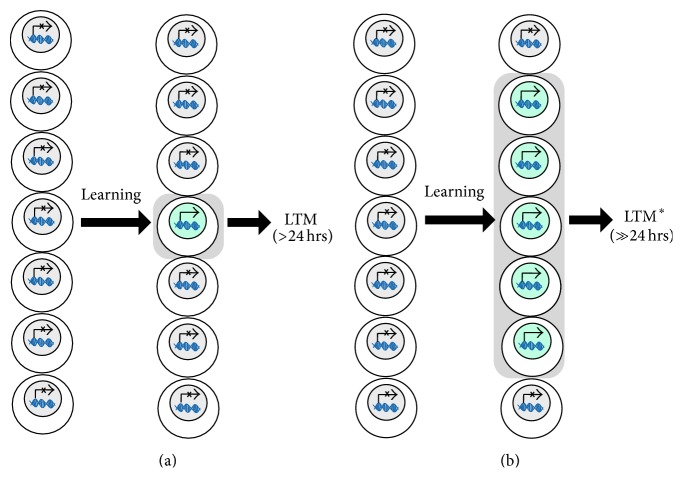
Long-term memory formation at a systems level.* A schematic showing how a learning event can lead to two different outcomes for long-term memory when considering populations of neurons.* (a) Learning processes can target a population of neurons that process task-dependent information during an experience. A typical threshold for long-term memory induction may only achieve gene expression events in a small number of neurons (here, only one out of the possible seven representatives of the population) that will undergo plasticity for memory consolidation and the formation of LTM. (b) If the threshold for memory induction is lower, gene expression events may occur in more neurons (here, five out of the seven neurons). The recruitment of more neurons with plasticity engaged could induce a larger network for memory that persists beyond typical long-term memory timescales. Notably, this conceptualization is applicable to any case of unusually strong and robust memories, including those induced by experimental manipulations (e.g., HDAC inhibition) and environmental influences such as stress, disease, drugs of abuse, or even therapeutic cognitive training paradigms.* Conventions are as in Figure 1*.

Despite these tantalizing findings, there remains the fundamental issue related to the much broader question in neuroscience about the nature of memory:* How is memory encoded to last even as long as a lifetime?* While exploring all possible answers to this key question is beyond the scope of this review, we propose that the conceptual platform for understanding behavioral epigenetic influences on the formation of lasting memory needs to expand beyond a traditional linear view of a simple conversion from short- into long-term memory. Indeed, the linear view of STM conversion to LTM is likely not complete since unique and nonoverlapping molecular profiles and their temporal constraints can be used to characterize and distinguish memory at different time scales (e.g., short, intermediate, and long) [25–34]. The current explanation has its critical factor at the neuronal level, which may operate in the timing and coincident expression of genes that produce gene products to assimilate neuronal effects into lasting effects on synaptic plasticity, circuitry, and behavior. Yet there is an equally compelling possibility complementary to neuronal-level and circuit-level models. That is to consider the systems-level effects of releasing the brakes on gene expression in populations of neurons (Figure 2(a) versus Figure 2(b)). In the following sections, we will consider this alternative “systems-level” approach.

## 4. A Potential Neural Correlate for the Robust Strength of HDACi-Enabled Memories

The known effects of HDAC inhibition on memory strength could be explained using an information encoding perspective that utilizes a systems-wide level of analysis. Taking examples from the ORM and OLM tasks described above, the necessary basis of recognition during long-term retention tests after 24 hours, at multiple days, or even longer after training requires memory for information about features specific to the objects (e.g., identifying features, like odor, color, or location). The neural substrate that enables the multitude of sensory information necessary to identify “an object” or “a place” that has been encoded into LTM might exist within and between a network of neurons that represent those features. Studies like that of McQuown et al. [13, 19, 43, 44] show brain region specificity of epigenetic function, for example, by inhibiting HDAC3 in neurons that process the information to be remembered to enable hippocampal plasticity for subsequent location-specific memory and behavior. Thus, HDACs may likewise enable the efficacy of transforming information from short-term to long-term memory stores by permitting gene expression that engages plasticity in a local group or widespread population of neurons that are sensitive to the various pieces of task-relevant information (Figure 2(b)).

Furthermore, the strength of long-term memories—particularly those enabled by HDAC inhibition—might be explained by the greater amount of information that becomes encoded into memory. This idea is in agreement with the suggestion that epigenetic function acts beyond the mere linear progression of information encoding from short-term into (via gene expression) long-term memory at the neuronal level. A complementary mode of HDAC function is likely to be systems-wide by engaging multiple events of gene expression underlying plasticity in various specific populations of neurons (Figure 2). Therefore, the overall effect of HDAC inhibition could be retaining more information about a learning experience, which is accomplished by engaging more neurons where plasticity subsequently ties them together to participate in the total network of memory.

## 5. Information Encoding by the Plasticity of Sensory Cortical Neurons

In sensory cortex, “where” or “in which neurons” plasticity occurs can directly influence the information that is learned and remembered, including also* how much* information is encoded [9, 45–49]. Sensory cortex with its native hierarchical organization—receptive fields (RFs) for neural “tuning” and their overlying cortical representations, for example, topographic maps—can distinctively code for precise information that is sensed, learned, and remembered. Upon this cortical map, each sensory neuron is analogous to a pushpin, which marks distinct and discrete representations of the external sensory world that have learned behavioral relevance. Epigenetic mechanisms like the HDAC3 molecular brake exploit these unique identifiers to selectively induce a memory for behaviorally relevant sensory cues. Just as HDAC3 can have action in hippocampal neurons to influence location memory without effects on features of memory independent of the hippocampus (e.g., for objects* per se*), HDAC3 might have action in sensory neurons that represent one sensory cue without effects on the multitude of nonrelevant sensory features of stimuli encountered in the totality of a typical perceptual experience.

**Figure 3:**
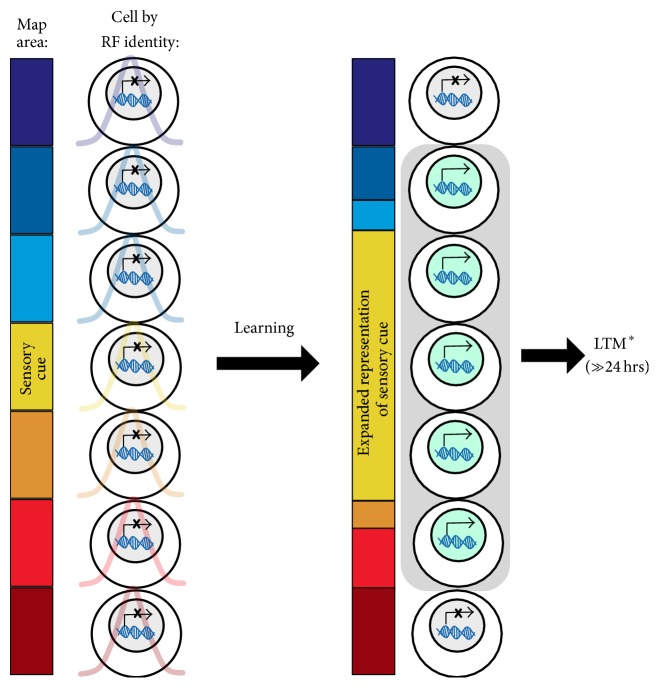
Strong and specific long-term memory formation via sensory cortical neurons.* Applying a systems-level approach to model epigenetic influences on the robustness of memory in a sensory cortex.* Distinct and discrete representations of the external sensory world (color heat map,* left panel*) are mapped onto cortical areas bounded by similar receptive fields (RFs) that reveal the neural “tuning” identity for sensory signals (colored curves underlying each neuron,* left panel*). As per the conceptualization described in Figure 2, learning events can target a selected subset of sensory neurons that have neural tuning to the various sensory cues available during an experience. Of those targeted, a lowered threshold for memory induction will facilitate specific memory for the behaviorally relevant sensory cues and features represented by neurons tuned to those features. These neurons would therefore be engaged with gene expression for subsequent neural plasticity. The right panel depicts one possible outcome for sensory cortical map plasticity: an enlarged representation of a behaviorally significant sensory cue (color heat map,* left panel*). For example, the magnitude of tone-frequency expansion in the frequency map of A1 has been shown to directly relate to the specificity and strength of auditory memory: more cells, then stronger memory [14, 15]. The general outcome of this framework is for more sensory information to be encoded by the activity and plasticity of those neurons tuned to the behaviorally relevant stimuli, which ultimately results in a robust memory formation.

In the laboratory, the HDAC3 “molecular brake” can be selectively removed via pharmacological manipulations. But real-world experiences might naturally invoke mechanisms that remove the brake in distinct populations of neurons that are uniquely tuned to the behaviorally relevant sensory cues of a learning experience (e.g., for location in hippocampal neurons). The resultant lowered threshold for memory consolidation creates a permissive state of gene expression to produce plasticity only in neurons that represent the significant stimulus feature(s) and not in other neurons representing irrelevant stimulus features. Furthermore, this predicts that experiences that induce more sensory neurons with their molecular brakes disengaged will result in more sensory information encoded by the activity and plasticity of those neurons. Together, this group of neurons ultimately participates in the network of the newly formed memory (Figure 3). In cases of object recognition, as in the tasks described by Stefanko et al. [12] and McQuown et al. [13], we predict that sensory cortical neurons whose responses are sensitive to the collection of identifying features of an object (like odor, color, texture, etc.) became engaged for long-term plasticity with HDAC inhibition. The network of sensory neurons engaged during initial experience captures both the task-dependent features and the collective information about object attributes that could subsequently support discriminative memory for one object over another. This suggests that when the network is made more extensive by releasing molecular brakes in more sensory neurons, there is an increased likelihood of encoding discriminative features of similar objects that would promote performance in a subsequent memory test.

Therefore, in addition to epigenetic mechanisms altering the threshold for transforming short-term into long-term memory stores, we here suggest a complementary hypothesis that epigenetic mechanisms can alter the breadth of information captured by engaging plasticity in selected brain regions and populations of neurons with distinct neural representations of sensory information. This idea is a natural corollary of the “informational capture” hypothesis first introduced by Bieszczad et al. [9], suggesting that HDACs are molecular brakes normally engaged to* prevent* all perceptually available information to become encoded into memory. Indeed, natural memory is selective under normal conditions. The unusual longevity of LTM formed by, for example, blocking HDAC3 could be explained by the release of the normal brakes placed on the susceptibility of the brain to encode more or even all available sensory information.

## 6. Molecular Brakes on Learning-Induced Plasticity of Sensory Cortical Neurons

We will use an example in the primary auditory cortex (A1) to make predictions from the hypothesis that HDAC functions in the formation of robust long-term memories. A large body of literature documents how experience-dependent sensory cortical plasticity underlies sensory-cognitive functions like sound signal detection, auditory discrimination, identification, and, importantly, memory formation (see for review [50–52]). A common theme of neuroplasticity in A1 appears to be that important sounds have enhanced representation in cortical receptive fields and maps. Various forms of A1 plasticity appear to remodel cortical auditory maps in an experience-dependent way (see for review [53, 54]). Specific signals can have enhanced representations via signal-specific map expansions [45, 53–57] or contractions [58, 59], evoked threshold shifts [60, 61], and receptive field bandwidth alterations [55, 62–64].

Auditory memories are selective for the significant and behaviorally relevant acoustic features of auditory learning experiences. Learning can attribute significance to formerly arbitrary sounds that have new acquired value to signal desired (appetitive) [57, 65] or detested (aversive) events (e.g., [66, 67]) or socially salient vocal communications [68–70]. A sound with acquired significance can remodel A1 as receptive field changes that can also accumulate to expand representations of that sound in the cortical map. The representative location and magnitude of receptive field changes for auditory cortical map expansion provide opportunities to encode specific information about learned sounds by selecting (1) in which neurons and (2) what representational form of plasticity those neural responses will undergo [45, 57, 66, 71–73]. This allows the brain a systematic way to encode any of many acoustic features significant to an auditory experience into a newly formed memory [74]. Moreover, evidence is accumulating that the magnitude and signal area of learning-induced tonotopic map expansion appear to enable both the strength of auditory memory formation and the specificity for what sounds are encoded into memory [14, 45, 57, 66, 71–73]. Interestingly, this system can also create “false” memory for sound signal identity when map expansions are artificially induced using brain stimulation techniques [14, 75, 76]. Thus, A1 plasticity itself might underlie the actual (though not necessarily veridical) specificity and strength of acoustic information encoded from experience into newly formed memory.

An emergent question is how auditory cortical neurons become selectively and differentially engaged for plasticity. Such a selective engagement of neural plasticity is where epigenetic mechanisms might come into play. For example, an HDAC molecular brake engaged in sensory cortical neurons would reduce the likelihood that the neurons would be recruited for experience-dependent cortical remodeling after being activated during perception. Lesburguères and colleagues [77] used an olfactory associative learning task to identify the fact that long-term associative memory involved an early “tagging” of selective cortical neurons by hippocampal-cortical interactions. Epigenetic mechanisms at these sites were discovered to alter neuronal function for subsequent memory consolidation. These data provide evidence of within-region selectivity for epigenetic mechanisms to engage (or disengage) in cortical neuronal populations. Furthermore, the experiments of Lesburguères et al. offer plausibility of neuronal tagging for subsequent long-term memory in the sensory cortex. The findings were of epigenetic “tags” in orbitofrontal cortical neurons that have a privileged role in processing the relevance of odor information [77].

## 7. Evidence for Behavioral Specificity of Learned Information under Epigenetic Control

Recently published behavioral evidence supports the idea that epigenetic regulation is a mechanism to control the specificity of learned information and dictate the sensory cues that are later remembered. For example, efforts from separate investigators using different species and sensory modalities have shown that DNA methylation can alter sensory discrimination behavior (e.g., for auditory cues in rats [10] and olfactory cues in honeybees [11]). We highlight the relevant findings here.

Using a standard associative learning paradigm, Biergans et al. [11] conditioned honeybees to associate an odorant (conditioned stimulus, CS) with a sucrose reward. They assessed the effects of DNA methyltransferase inhibition on long- and short-term memory formation in honeybees, by quantifying memory retrieval as a function of proboscis extension response to the CS odor relative to its response to a new odorant across three discrete time points (30 minutes, 1 day, and 3 days). Bees were treated either with the DNA methyltransferase inhibitor zebularine in solution or with solvent-solution alone. Learning rates during the conditioning procedure and test for memory strength at retrieval did not differ between these two groups. However, the authors measured as an index of olfactory discrimination the difference between the proboscis extension response to the CS and the new odorant. Interestingly, only in the 1- and 3-day memory retrieval test was the memory discriminatory power significantly larger in the solvent-treated group than in the zebularine group. The authors concluded that DNA methyltransferases are not involved in short-term memory formation in honeybees (as measured by the 30-minute time point). In contrast, DNA methylation was necessary for mediating the olfactory discriminatory power of long-term memory (measured days later).

Histone acetylation has likewise been reported to control LTM formation in classically conditioned honeybees, which posits a general rule that epigenetic mechanisms are key and conserved regulators of neuroplasticity underlying perceptual and cognitive behaviors [78, 79].

To address stimulus specificity mediated by DNA methylation in rodent associative reward learning, Day et al. [10] investigated its role to mediate neuroplasticity in the reward circuits of the brain that could underlie changes in reward-directed behavior. Experience-dependent changes due to associative learning were differentiated from those arising due to the reward itself or environmental experiences alone by training separate groups of rats in three different Pavlovian sound-to-reward conditioning paradigms [80]. All paradigms used the same auditory signal cue, but that cue was either predictive of a sucrose reward (CS+), explicitly unpaired (CS−) with sucrose reward, or used in conjunction with exposure (CS_0_) to the conditioning chamber without reward delivery. In a series of elegant experiments, the authors showed that only the CS+ group exhibited reward-related memory formation. Moreover, learning about the CS+ was the only condition with selectively increased expression of the immediate early genes* egr1* and* fos* in the ventral tegmental area (VTA) thought to be essential for reward-related learning [81]. Critically, these neurons only in the CS+ conditioned animals were also reported to have activity-dependent DNA methylation.

Together, these experiments support the hypothesis that epigenetic mechanisms have a role in the sensory specificity of remembered events. However, these data only describe the behavioral evidence of epigenetic influences. Previous findings from the field of sensory behavioral neuroscience link sensory specific memory strength with cortical plasticity (e.g., [55]) and lead to the possibility that behavioral specificity of information about the remembered sensory cues and features has anatomical substrates in the experience-dependent plasticity of the sensory cortex.

Bieszczad et al. [9] directly tested whether epigenetic mechanisms can alter sensory cortical plasticity and behavioral correlates of sensory information capture and storage. The authors applied an auditory model of learning, memory, and auditory cortical plasticity to investigate how histone acetylation might change information processing for* what* and* how much* becomes encoded into behavioral memory by selectively engaging A1 plasticity. Rats were treated with a class I HDAC inhibitor with enhanced selectivity for HDAC3 (RGFP966) while learning to associate the sound with reward in an auditory instrumental conditioning paradigm. These animals remembered the signal sounds with greater frequency-specificity relative to a performance matched vehicle control group [9]. Furthermore, the RGFP966-treated animals were able to encode additional auditory information about a second sound signal. Therefore, HDAC inhibition induced animals to remember more auditory information. Moreover, the greater behavioral specificity for the reward-related auditory cues in animals treated with RGFP966 was reflected in enhanced cortical representations of the remembered signal sounds in A1. Specific acoustic features of the sound signals predicting reward (such as acoustic frequency and sound loudness), and the additional memory for the second sound signal, all had enhanced representation in A1. In contrast, no vehicle-treated animals developed highly specific auditory memory, nor did they show significant cortical remodeling for the multiple sound signals associated with reward. The authors concluded that RGFP966 enabled the formation of a more specific and complex auditory memory that incorporated additional information about the behaviorally relevant features of sound. The neural basis of the richness of memory appeared to have been supported by unusually signal-specific remodeling of the auditory cortex.

## 8. Overall Conclusion

Overall, existing data illustrate that epigenetic function in learning and memory processes both physiologically and psychologically is compatible with the idea that epigenetic mechanisms could control “informational capture” at a systems level. Here, we specify that the plasticity of the sensory cortex may be an essential part of the behavioral epigenetic influence on long-term memory formation. We highlight that class I HDACs (and maybe in particular HDAC3) are especially important to gate sensory cortical plasticity that underlies the sensory complexity of newly formed memories during memory consolidation by enabling “what” and “how much” information becomes encoded. In turn, the sensory richness of memory enabled by sensory cortical plasticity may be at the root of the robustness of some long-term memories, which survive even after the passage of time or with interfering experiences.

The proposed systems-level substrate for unusually strong memory induced by experimental manipulations of epigenetic function (e.g., HDAC inhibitors, DNA methyltransferase inhibitors or activators) need not be limited to memory formation that is adaptive* per se*. For example, a sensory cortical substrate of epigenetic influences that engages more neurons in neuroplasticity (including more sensory regions and other modalities) might explain both the intrusiveness and the robustness of emotional memories [20], or stress related memories, including that of trauma [82] and drug addiction [83]. If such experiences naturally elicit epigenetic changes that mimic the enabling effects of the experimental interventions described, then the brakes on plasticity would likewise be released to incorporate more sensory neurons—and, thereby, more sensory information—into memory. This expanded network of sensory information underlying the newly formed memory would not only make these experiences more difficult to forget, but the increased number of involved cells would be more likely to activate underlying networks for memory retrieval during spontaneous activity or a related experience that would otherwise be below the threshold for reactivation.

## 9. A New Direction for Future Research

This paper has surveyed a body of work that utilized either acoustic stimuli (pure tones, artificial stimuli) or object identifiers (location, odor, and identity) in animals to reveal how epigenetic mechanisms might enable encoding of highly selective and specific sensory information from these experiences into robust and lasting memory. Applying the hypothesis of a novel epigenetic mechanism for specific, selective, and strong encoding of sensory information could inform a new avenue for discovery in the domain of vocal communication learning, where learning about the precise sounds, their relevant acoustic features, and behaviorally relevant significance is absolutely essential. Vocal communication learning is an excellent model with which this hypothesis can be tested. For example, we predict that epigenetic control of transcription could facilitate changes in neuronal function that regulate the learning and production of communication signals in the songbird, an established animal model of vocal learning and memory.

Songbirds learn their vocal communication signals much as humans do and provide a large repertoire of behaviorally significant signals that can be used as unique stimuli in invasive physiological experiments where well-documented forms of natural sensory and sensory-motor learning provide special experimental and conceptual advantages [84–87]. Initial experiments could be with HDAC-targeted techniques. Advantageously, the HDAC3 sequence in the avian brain is directly homologous to that in mammals, which provide feasibility of testing currently available HDAC3-selective pharmacological inhibitors in birds. In a further parallel, specialized avian brain regions exhibit neural responses that show the essential features of higher-level auditory perceptual and cognitive functions: enhanced neural discrimination among similar acoustic vocalization sounds and signal-specific neural plasticity reflecting memory for recently heard significant sounds. These functional properties of the avian auditory brain make this system a prime model for understanding the neural bases of human speech processing, language acquisition, and speech comprehension. Future research could also extend to developmental models if epigenetic mechanisms are found to control the ability of juveniles (like infant humans) to acquire and store unlimited amounts of detailed acoustic information necessary for language acquisition by having molecular brakes disengaged to allow this flood of information to become encoded, for example, during critical periods until adulthood [88, 89]. If this is the case, then a pharmacological HDAC inhibitor has the potential to transcend into clinical research for its efficacy to reenable the ease of auditory memory formation in adult, aged, or diseased brains that have otherwise lost the essential functions for speech and language learning and comprehension.

Moving forward, it will be necessary to keep in mind the multiple levels at which behavioral epigenetics exert influences (neuronal, circuit, and systems) to ultimately control behavior. The sensory cortices are now a fertile ground for discovery to understand how epigenetic mechanisms contribute to information processing that enables the combined perceptual and cognitive function of remembering the specific and select sensory content of important experiences.
